# Contribution of Fourier transform infrared spectroscopy for outbreak investigation of carbapenem-resistant *Acinetobacter baumannii*

**DOI:** 10.1128/spectrum.02392-25

**Published:** 2025-12-19

**Authors:** Hadas Kon, Mor N. Lurie-Weinberger, Dafna Chen, Hani Laderman, Elizabeth Temkin, Elena Lomansov, Ibraheem Firan, Worood Aboalhega, Marina Raines, Alona Keren-Paz, Yehuda Carmeli

**Affiliations:** 1 National Institute for Antibiotic Resistance and Infection Control, Tel Aviv, Israel; 2 Infection Control Unit, Rambam Health Care Campus 58878, Haifa, Israel; 3 Faculty of Medical and Health Sciences, Tel Aviv University 26745, Tel Aviv, Israel; Johns Hopkins University, Baltimore, Maryland, USA

**Keywords:** single nucleotide polymorphism (SNP) threshold, whole genome sequencing, carbapenem-resistant *Acinetobacter baumannii*, outbreak, fourier transform infrared spectroscopy

## Abstract

The value of Fourier transform infrared (FTIR) spectroscopy as an investigation tool for outbreaks caused by *Acinetobacter baumannii* remains inconclusive. Here, we used FTIR as a real-time tool to investigate a carbapenem-resistant *A. baumannii* (CRAB) outbreak and later compared the FTIR results to results of whole genome sequencing (WGS). We used single-nucleotide polymorphism (SNP) analysis to confirm the correct FTIR cutoff and validate FTIR clustering. The outbreak occurred mainly in a general intensive care unit of an Israeli tertiary care hospital and involved 22 patients detected between December 2022 and August 2023. Isolates from 21 patients and two environmental sources were analyzed by FTIR. Eleven representative isolates underwent WGS. We performed SNP analysis using the earliest isolate as the reference. FTIR revealed that the outbreak was comprised of three clusters and four singletons. FTIR results were concordant with WGS for the 11 sequenced isolates. FTIR had higher discriminatory power than multilocus sequence typing. Within-cluster SNP differences were up to 21 SNPs. This finding confirmed that the cutoff we chose to define FTIR clusters (0.252) was appropriate. FTIR uncovered that what appeared to be one CRAB outbreak was actually three parallel outbreaks. FTIR analysis of CRAB outbreaks yields results similar to WGS, but faster and at a lower cost. A SNP threshold of less than 21 SNPs is appropriate when analyzing an *A. baumannii* outbreak.

**IMPORTANCE:**

This study demonstrated that Fourier transform infrared (FTIR) spectroscopy could effectively identify *Acinetobacter baumannii* outbreaks. Serving as a practical and rapid alternative to whole genome sequencing, FTIR can significantly accelerate *A. baumannii* outbreak confirmation, resulting in effective infection control interventions.

## INTRODUCTION

Nosocomial outbreaks caused by carbapenem-resistant *Acinetobacter baumannii* (CRAB) are of high clinical relevance due to the organism’s environmental persistence and high associated mortality (1). These characteristics are facilitated by *A. baumannii*’s ability to survive on dry surfaces for up to 5 months (2) and its propensity to harbor multiple antibiotic resistance genes and virulence factors (3). *A. baumannii* can be transmitted via direct or indirect contact and frequently colonizes the skin and upper respiratory tract of hospitalized patients (4). Early and thorough investigation of CRAB outbreaks, including identification of the outbreak source and modes of transmission and molecular and genetic characterization of the isolates, is essential for implementing infection control measures to eliminate the bacterial reservoir and terminate the outbreak.

Whole genome sequencing (WGS) of isolates involved in an outbreak is the gold standard for typing, but it can be costly and time-consuming, and results may be delayed. Fourier transform infrared spectroscopy (FTIR) typing is a novel technique offering a rapid and cost-effective alternative. FTIR measures the absorption of infrared light by the molecules present in the bacterial cell, generating a unique spectrum that represents the isolate’s specific physicochemical content. Each spectrum is compared with all other spectra, and a dendrogram is constructed by calculating the distances between spectra according to hierarchical cluster analysis.

Here, we used FTIR as a real-time tool to investigate a CRAB outbreak and later compared the FTIR results to results of WGS. We used single-nucleotide polymorphism (SNP) analysis to confirm the correct FTIR cutoff and validate FTIR clustering.

## RESULTS

The hospital identified the outbreak’s index patient on 6 December 2022. CRAB screening upon admission to the intensive care unit (ICU) was not performed, and so the case was classified as hospital-acquired but may have been imported. CRAB cases continued to emerge in the ICU at an average rate of one case every 2 weeks but reached a peak in May with six cases. From December 2022 to the end of the outbreak in August 2023, 21 patients in the ICU (including the index patient) acquired CRAB: eight had CRAB isolated in clinical cultures, and 13 were detected by screening. In addition, one patient in the pediatric surgical ward with CRAB isolated from a wound was considered part of the outbreak because he shared a surgeon with a patient with CRAB in the ICU. This case was identified during a hospital-wide epidemiological investigation, conducted to identify additional CRAB cases during the outbreak period. Aside from this individual, no additional patients outside the ICU met criteria for inclusion in the outbreak based on spatiotemporal overlap or shared exposure to healthcare personnel.

The epidemiological investigation identified several factors contributing to the outbreak, including low compliance with hand hygiene and isolation protocols, lack of cohorting of CRAB carriers with dedicated staff, compromised ability to clean equipment properly due to wear and tear, and use of shared cleaning carts for CRAB carriers and non-carriers. Environmental sampling of frequently touched surfaces (*n* = 43) was performed in May: two of 43 samples were positive for CRAB, one from a wooden mop handle or cleaning supplies cart (the same swab was used to sample both sites) and one from a sink. Measures taken to control the outbreak included: staff education, cohorting of CRAB carriers and assigning dedicated staff to the cohort, discarding worn-out equipment, replacing all sink drains in patient rooms, reducing shared carts and equipment, enhancing cleaning routines, and replacing all wooden mop handles with washable metal handles.

**Fig 1:**
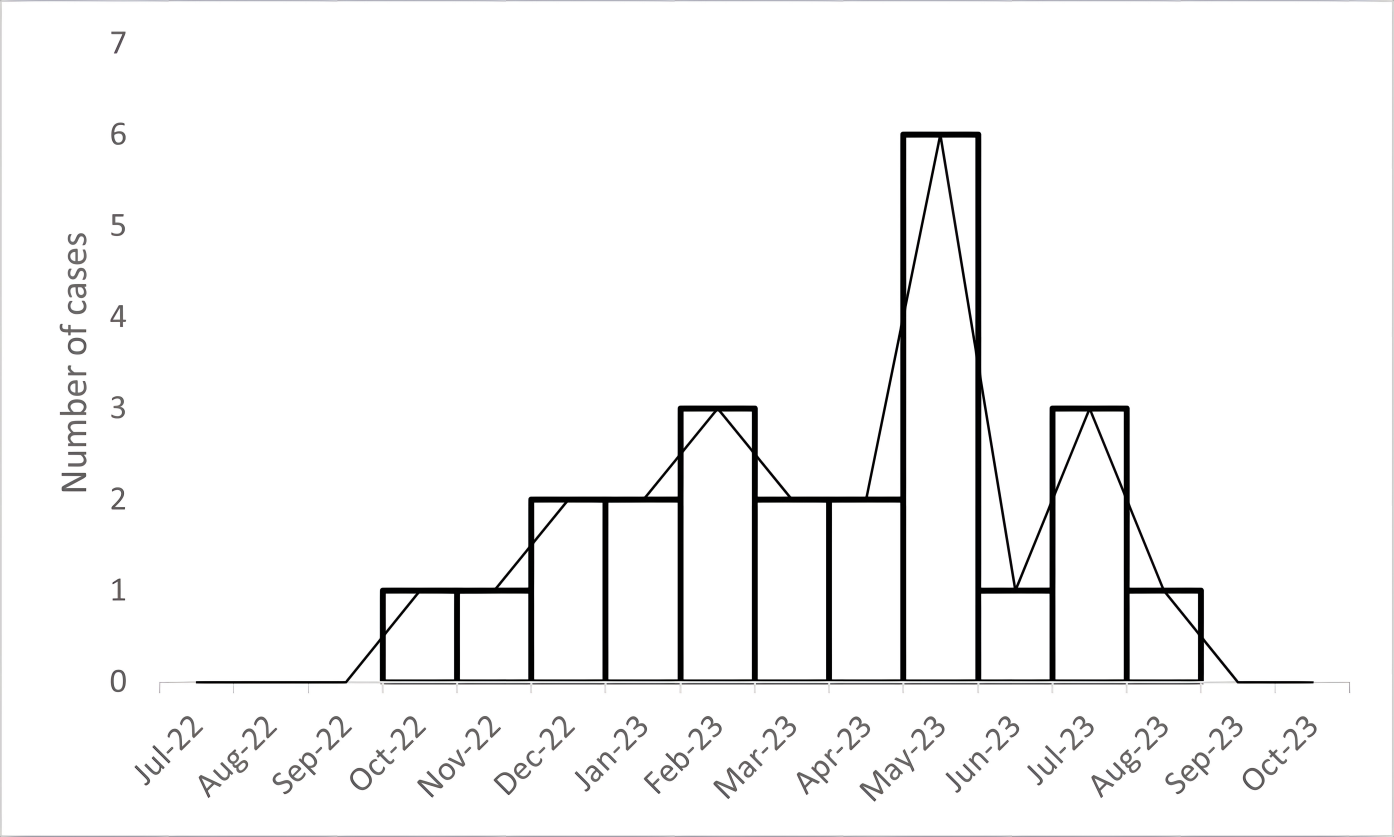
Epidemic curve. Numbers of individuals carrying CRAB, by month. The outbreak isolates tested in the study, composing 22 clinical isolates, are displayed between December 2022 and August 2023.

The epidemic curve is presented in Fig. 1. The CRAB outbreak included 22 clinical CRAB samples, with a peak of six clinical samples recovered in May 2023. Only two cases of CRAB were detected 5 months prior to the outbreak (Fig. 1).

**Fig 2:**
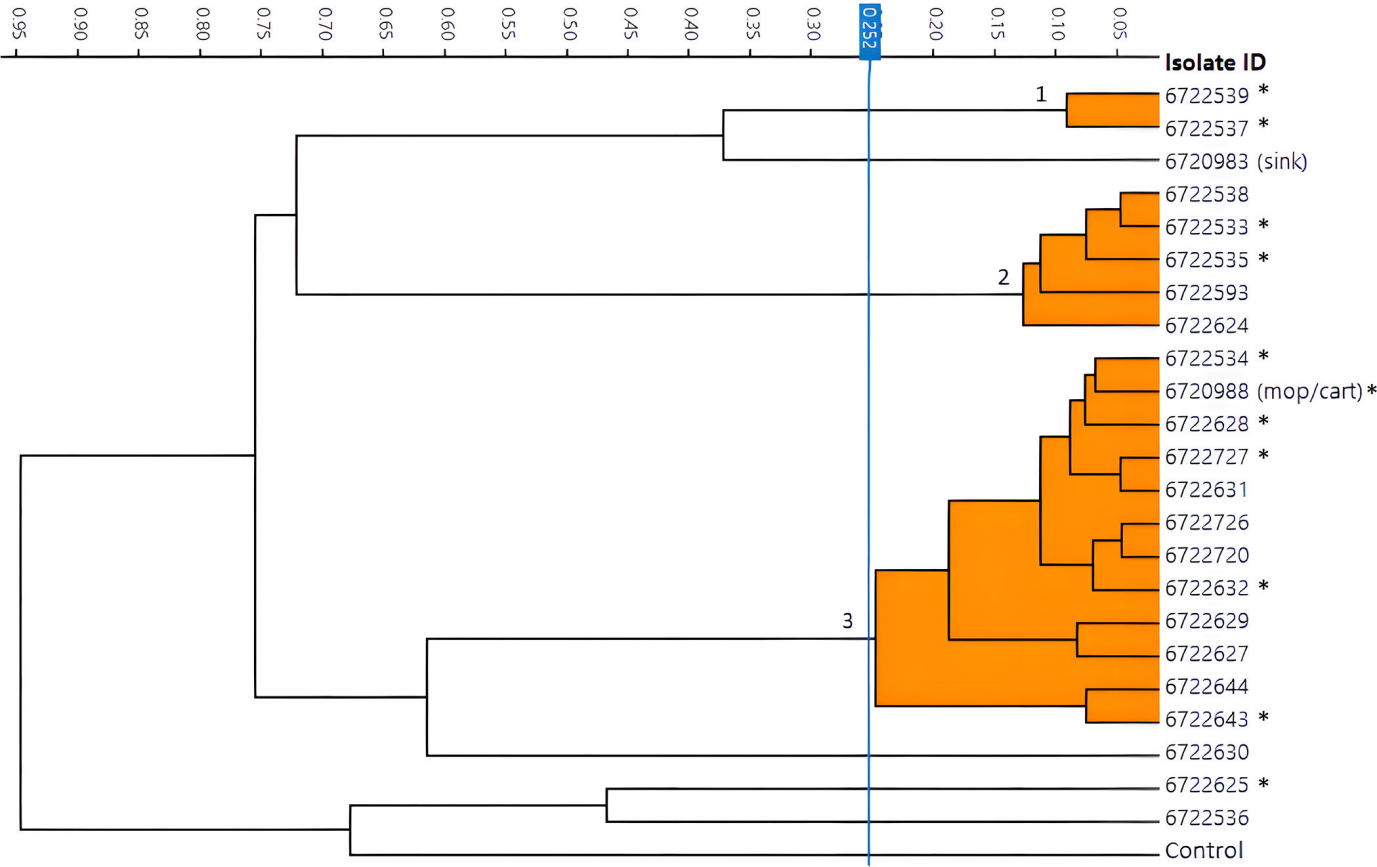
FTIR dendrogram of outbreak isolates showing three clusters. Sequenced isolates are denoted by an asterisk (*).

The FTIR dendrogram of the outbreak isolates revealed one large cluster of 12 isolates (cluster 3), two smaller clusters (clusters 1 and 2), and four singletons (Fig. 2). The environmental sample from the mop handle/cart (isolate ID 6720988) belonged to the large cluster; the sample from the sink (isolate ID 6720983) was not closely related to any other outbreak isolate. The epidemiological investigation revealed links between four patients (isolate IDs 6722727, 6722632, 6722726, and 6722720) in cluster 3 who had undergone X-ray examinations, indicating a common exposure from either the portable X-ray machine or the shared radiologic technologists. Moreover, the patient from pediatric surgery (isolate ID 6722535) and the ICU patient who had the same surgeon (isolate ID 6722533) belonged to the same cluster (cluster 2). No epidemiological link was identified among the singletons.

**Fig 3:**
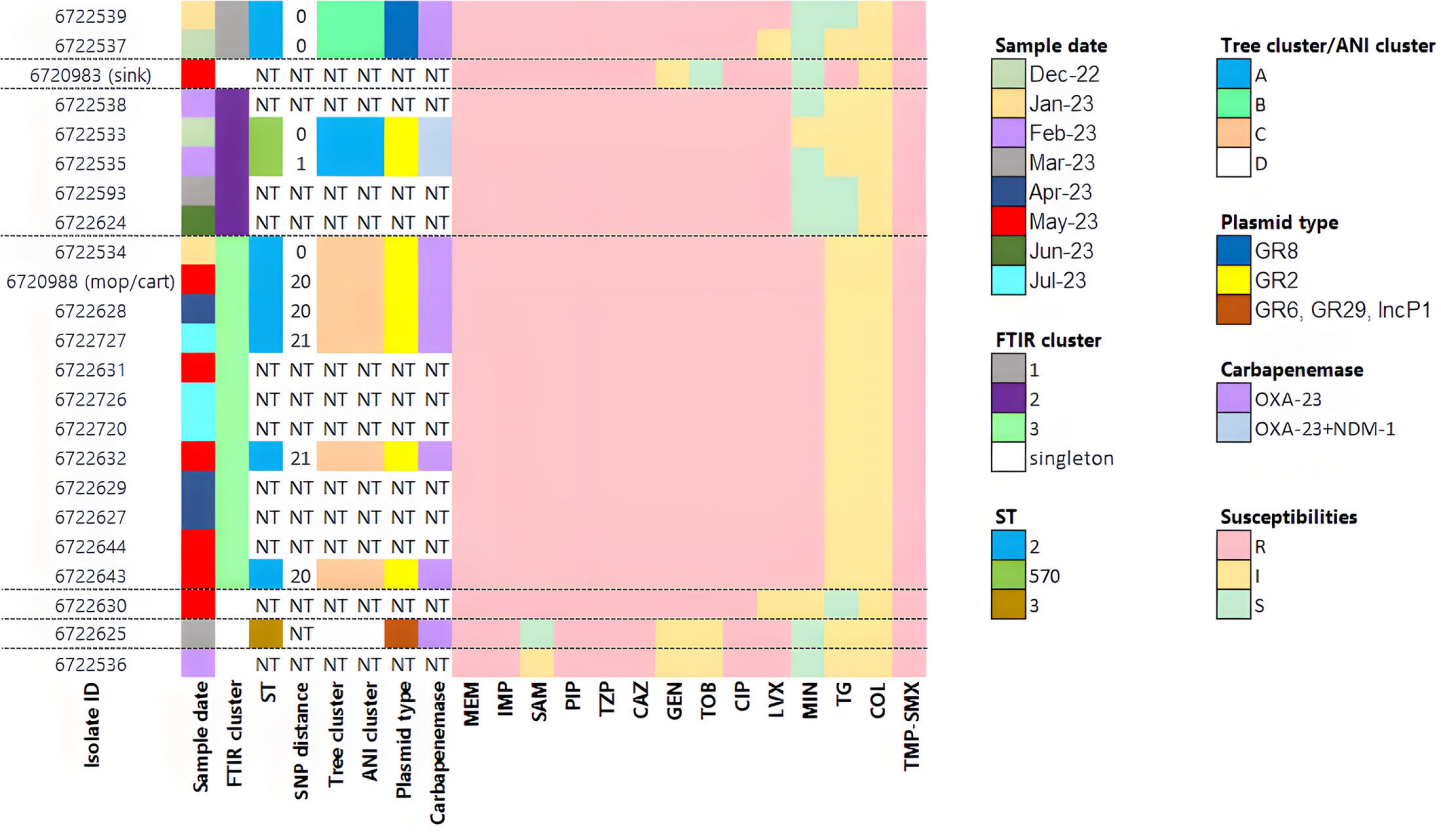
Heat map of the outbreak isolates. The dotted lines indicate the division according to FTIR clusters. Phylogenetic tree and ANI clustering was based on Fig. S1. NT, not tested; ST, sequence type; MEM, meropenem; IMP, imipenem; SAM, ampicillin-sulbactam; PIP, piperacillin; TZP, piperacillin/tazobactam; CAZ, ceftazidime; GEN, gentamicin; tobramycin, TOB; ciprofloxacin, CIP; LVX, levofloxacin; minocycline, MIN; tigecycline, TG; colistin, COL; trimethoprim/sulfamethoxazole, TMP-SMX.

FTIR results were concordant with WGS results for the 11 sequenced isolates (Fig. 3). Three different sequence types (STs) were identified: the two isolates in FTIR cluster 1 belonged to ST2, six isolates from cluster 3 also belonged to ST2, two isolates from cluster 2 belonged to ST570, and a singleton belonged to ST3. Cluster 3, the largest cluster, included patients detected over seven months. The SNP distance between isolates in the same cluster was up to 21 SNPs in cluster 3, one SNP difference in cluster 2, and in cluster 1, the isolates were genetically identical (0 SNPs). This finding confirmed that the cutoff we chose to define FTIR clusters (0.252) was suitable for the outbreak investigation. The phylogenetic tree and the ANI matrix grouped isolates into the same clusters as FTIR (Fig. 3; Fig. S1). As seen in Fig. 3, three plasmids were detected; two were cluster-specific and one crossed clusters. All isolates harbored *bla*OXA-23, with the coexistence of *bla*OXA-23 and *bla*NDM-1 in isolates belonging to cluster 2. The susceptibility profiles aligned with the FTIR and WGS results, with similar susceptibility patterns observed within each cluster. Where discrepancies existed—such as isolates within a cluster exhibiting susceptible versus intermediate results to a given antibiotic—they were a single doubling dilution apart and thus fell within the margin of error (Fig. 3).

## DISCUSSION

In this study, we described a CRAB outbreak and the use of FTIR typing as a tool to determine isolate relatedness. FTIR revealed that the outbreak was composed of three different clusters and four singletons. The detection of three simultaneous outbreaks using FTIR demonstrates its efficacy as an outbreak identification tool in high-prevalence settings. FTIR supported the hypothesis of cross-transmission within the ICU and spillage to a patient outside the ICU and provided insights on the relatedness between an environmental isolate (the mop handle/cart) and patients. The sink isolate could have been part of a cluster of two patients (sampled six months before the sink) if we had chosen a higher cutoff of 0.40.

FTIR has been applied for the discrimination of bacteria (5–7) and as a tool for outbreak investigations caused by various bacteria (8–10). Several studies proved FTIR to be a successful and accurate tool for analysis of outbreaks involving *A. baumannii* (8, 9, 11–13), but others reported unsatisfying FTIR results for *A. baumannii* outbreaks (14). Moreover, in these studies, FTIR results were either unvalidated (8, 11) or confirmed by methods less discriminatory than WGS, such as pulsed-field gel electrophoresis or multilocus sequence typing (MLST) (9, 12), with only one study providing WGS-level confirmation (via MLST and ANI) (13).

While a universal SNP threshold for CRAB has not been established, studies commonly employ a threshold of approximately 20 SNPs to identify outbreak clusters (15–17). Our results align with this practice, demonstrating that a threshold of fewer than 21 SNPs effectively differentiates non-outbreak from outbreak CRAB isolates.

A limitation of this study was the inability to perform WGS on the sink sample, as the isolate could not be grown. This absence of sequencing data, combined with a lack of epidemiological links, prevented us from raising the FTIR cutoff value; therefore, we employed a lower, more conservative cutoff. An additional limitation was that the outbreak occurred in a single hospital unit. Future studies should include a more diverse and representative set of isolates from multiple units and institutions to better assess the broader applicability of FTIR in CRAB outbreak investigations.

In conclusion, our FTIR analysis suggested that an apparently single epidemiological outbreak was actually composed of three distinct clusters. This provided us the opportunity to examine the discriminatory power of FTIR. To do so, we compared the results of FTIR clustering with MLST typing and with SNP analysis. We demonstrated that FTIR had higher discriminatory power than MLST. SNP analysis confirmed that the clustering identified by FTIR was correct. (Because the sink isolate was not sequenced, we could not verify the appropriateness of using the higher FTIR cutoff of 0.40). In addition, based on the results observed in this study, a threshold of less than 21 SNPs is appropriate when analyzing an *A. baumannii* outbreak.

## MATERIALS AND METHODS

### Setting

The CRAB outbreak occurred in the general ICU of a tertiary-care hospital in Israel, from December 2022 to August 2023. In this hospital in 2023, the annual incidence of hospital-acquired CRAB per 100,000 hospital days was 16.6 in clinical cultures and 5.1 in blood. The ICU has 18 beds in single or multi-patient rooms with curtains between patients. The ICU’s CRAB surveillance policy included screening of the rectum and throat upon admission and once a week thereafter if there were any known CRAB carriers in the unit. Additional screening sources were tracheal aspirate in ventilated patients and skin in patients who were contacts of a CRAB carrier.

### Isolates

CRAB isolates from 21 patients and two environmental sources were shipped to the reference laboratory; no isolate was shipped for one patient. Isolates were grown on selective agar (modified CHROMagar Acinetobacter, containing 4.5 mg/mL meropenem, HyLabs, Rehovot, Israel) and identified to the species level using VITEK MS (GN card) (*bioMérieux* SA, Marcy l’Etoile, France). Antibiotic susceptibility was determined using VITEK 2 (*bioMérieux*).

### FTIR typing

FTIR typing was performed according to the IR Biotyper (Bruker, Leipzig, Germany) manufacturer’s instructions. Briefly, isolates were grown for 24 hours on 5% sheep blood in tryptic soy agar (HyLabs, Rehovot, Israel) at 35±2°C, and samples were loaded on a 96-spot silicon sample plate (Bruker) in quadruplicate. Spectra were analyzed using hierarchical cluster analysis with Euclidean distance and average linkage. A dendrogram was generated by OPUS 7.5 software (Bruker) and was analyzed in the polysaccharide region (800–1,300 cm^−1^), as recommended by the manufacturer. A randomly selected, unrelated CRAB isolate served as a control. Quality control was performed according to the manufacturer’s recommendations and based on several criteria: absorption (0.4 arbitrary units [AU] < *D* value < 2 AU), noise (<150 × 10^−6^ AU), presence of water (<300 × 10^−6^ AU), and fringes (<100 × 10^−6^ AU). Samples that did not meet the quality control criteria were excluded from the analysis. Following inspection of the resulting dendrogram, we selected the cutoff value of 0.25, the most conservative cutoff option recommended by the manufacturer for *A. baumannii* (0.25–0.4). We examined the appropriateness of this cutoff by SNP analysis, as described below.

### WGS and bioinformatics analysis

We selected 12 isolates for WGS representing the various clusters and singletons identified by FTIR. The sink isolate could not be grown and therefore only 11 isolates were sequenced. Whole genomic DNA was extracted from a loop of overnight colonies re-suspended in 100 µL phosphate buffered saline. A total of 90 µL bacterial lysis buffer and 10 µL Proteinase K (Qiagen GmbH, Germany) were added, incubated for 10 min at 65°C then 10 min at 95°C. High-molecular-weight DNA was isolated using the MagLEAD 12gC (PSS co.) and MagDEA DX Sv Kit, according to the manufacturer’s instructions. Genomes were sent to Plasmidsaurus (Eugene, OR, USA) for Illumina sequencing. Isolates were annotated using Prokka and typed using PubMLST according to the Pasteur scheme. Antibiotic resistance genes were detected using ResFinder version 0.7.1. Plasmid GR groups were determined by BlastN versus a reference of GR types and by PlasmidFinder (https://cge.food.dtu.dk/services/PlasmidFinder/). Pan-genome analysis of the 11 genomes was constructed using Roary version 3.12.0 with an unrelated CRAB isolate as a control. A phylogenetic tree was constructed using RAxML version 8.2.12 with the GTRGAMMA model. SNP and average nucleotide identity (ANI) analysis was performed using CLC workbench version 25.0.2. To determine the genetic distance between isolates in the same FTIR cluster, SNP analysis was carried out using the earliest isolate as the reference. Similarly, distance between clusters was determined using a single common reference.

## Data Availability

The genomes were deposited in the BioProject database under the accession number PRJNA1172467.

## SUPPLEMENTAL MATERIAL

Figure S1 spectrum.02392-25-s0001.pdf Genetic relatedness among the outbreak isolates.

ASM does not own the copyrights to Supplemental Material that may be linked to, or accessed through, an article. The authors have granted ASM a non-exclusive, world-wide license to publish the Supplemental Material files. Please contact the corresponding author directly for reuse.
