# Supplementary material for: Contribution of Fourier transform infrared spectroscopy for outbreak investigation of carbapenem-resistant Acinetobacter baumannii
Source: Microbiol Spectr. 2025 Dec 19;14(2):e02392-25. doi: 10.1128/spectrum.02392-25 (PMC12889137; doi:10.1128/spectrum.02392-25)
Supplement: Figure S1 — Genetic relatedness among the outbreak isolates. [file spectrum.02392-25-s0001.pdf]

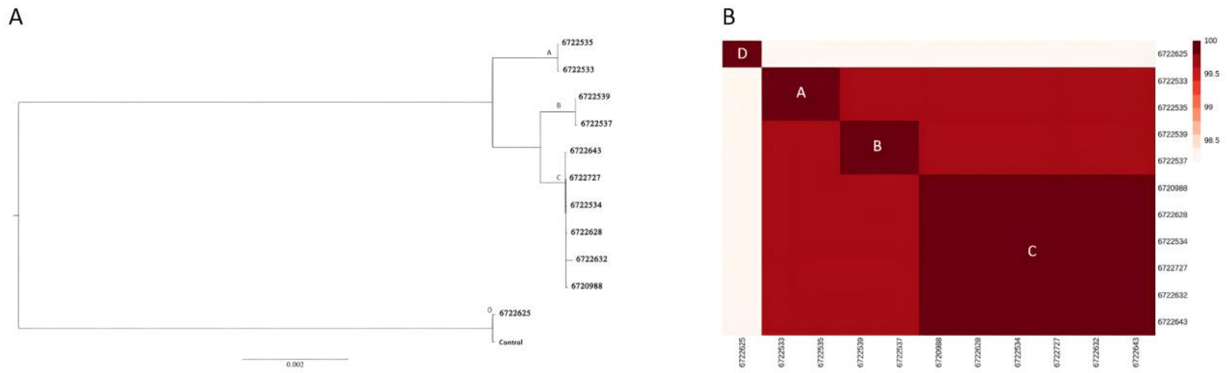

Supplement Figure S1. Genetic relatedness among the outbreak isolates. (A) Phylogenetic tree. Scale bar: 0.002 nucleotide substitutions per site. (B) Average nucleotide identity (ANI) matrix. Color scheme varies from high similarity (red) to low similarity (white) of the genomes analyzed.
